# Study protocol: the effect of vitamin D supplements on cardiometabolic risk factors among urban premenopausal women in a tropical country - a randomized controlled trial

**DOI:** 10.1186/1471-2458-13-416

**Published:** 2013-05-01

**Authors:** Mazliza Ramly, Foong Ming Moy, Rokiah Pendek, Suhaili Suboh, Alexander Tan Tong Boon

**Affiliations:** 1 Department of Social and Preventive Medicine, Faculty of Medicine, University of Malaya, Kuala Lumpur, 50603, Malaysia; 2 Julius Centre University of Malaya, Department of Social and Preventive Medicine, Faculty of Medicine, University of Malaya, Kuala Lumpur, 50603, Malaysia; 3 Department of Medicine, Faculty of Medicine, University of Malaya, Kuala Lumpur, 50603, Malaysia

**Keywords:** Vitamin D, Cardiometabolic risks, Quality of life, Premenopausal women

## Abstract

**Background:**

Besides its classical role in musculoskeletal diseases, vitamin D deficiency has recently been found to be associated with cardiometabolic risks such as hypertension, diabetes mellitus and hypercholesterolemia. Although Malaysia is a sunshine-abundant country, recent studies found that vitamin D deficiency prevalence was significantly high. However, few published studies that measured its effect on cardiometabolic risk factors were found in Malaysia. There are also limited clinical trials carried out globally that tried to establish the causality of vitamin D and cardiometabolic risks. Therefore, a double blind, parallel, randomized controlled trial on vitamin D and cardiometabolic risks is planned to be carried out.

The objective of this study is to investigate whether vitamin D supplements can reduce the cardiometabolic risk and improve the quality of life in urban premenopausal women with vitamin D deficiency.

**Methods/design:**

Three hundred and twenty premenopausal women working in a public university in Kuala Lumpur, Malaysia will be randomized to receive either vitamin D supplement (50,000 IU weekly for 8 weeks and 50,000 IU monthly for 10 months) or placebo for 12 months. At baseline, all participants are vitamin D deficient (≤ 20 ng/ml or 50 nmol/l). Both participants and researchers will be blinded. The serum vitamin D levels of all participants collected at various time points will only be analysed at the end of the trial. Outcome measures such as 25(OH) D3, HOMA-IR, blood pressure, full lipid profiles will be taken at baseline, 6 months and 12 months. Health related quality of life will be measured at baseline and 12 months. The placebo group will be given delayed treatment for six months after the trial.

**Discussion:**

This trial will be the first study investigating the effect of vitamin D supplements on both the cardiometabolic risk and quality of life among urban premenopausal women in Malaysia. Our findings will contribute to the growing body of knowledge in the role of vitamin D supplements in the primary prevention for cardiometabolic disease.

**Trial registration:**

ACTRN12612000452897

## Background

Vitamin D or also known as the “sunshine vitamin” is a fat-soluble vitamin that is associated with bone health. It is well recognized that vitamin D deficiency leads to rickets in children and osteomalacia and osteoporosis in adults [1]. However, recent findings have recognized unknown roles of vitamin D such as in the renin production in the kidney [2-4], insulin production in the pancreas [5-7], production of cathelecidin in macrophages [8,9] and growth and proliferation of both vascular smooth muscle cells and cardiomyocytes [7]. With these brand new discoveries, vitamin D maybe be associated with diseases such as hypertension, diabetes mellitus, hyperlipidemia and cardiovascular diseases [1,9-11].

A few observational studies have shown significant inverse correlation between blood pressure and renin-angiotensin activity [2,12-15]. An observational study in Korea also found a strong inverse association of vitamin D and hypertension [16].

Low serum vitamin D level has been proven to be inversely correlated with type 2 diabetes mellitus. It is known that normal insulin secretion in pancreatic ß-cells depends on vitamin D. A reduction in vitamin D level can result in an increase in insulin resistance and reduction in insulin secretion [2,6,9]. The prevalence of type 2 diabetes mellitus among South Asian origin women living in New Zealand with lower serum Vitamin D is three times higher than the New Zealand national incidence for diabetes [16].

Other mechanisms by which vitamin D may protect against cardiovascular disease is through decreased levels of triglycerides and low-density lipoprotein cholesterol. This is possible as vitamin D increases the lipoprotein lipase activity in adipocytes [17]. In addition, improving vitamin D levels will reduce the risk of developing type 2 diabetes mellitus and this will further reduce the lipoprotein disorders thus reducing the risk of cardiovascular diseases [18].

With these new discoveries, vitamin D deficiency could be considered as a new modifiable risk factor for cardiometabolic disease; however, this needs more persuasive evidence. Many observational studies indicate the association between vitamin D and cardiometabolic risks [10,17,19]. However, very limited trials are available to provide stronger evidence on the relationship between vitamin D and cardiometabolic risks. To date, only few clinical trials investigate cardiometabolic risks as its primary outcomes [14,16,20-23]. The results of these clinical trials are also often inconsistent in the effects of vitamin D supplementation on cardiometabolic risks, especially among the small trials [14,16,17,21,24-26]. Therefore, there is a need to determine whether treating vitamin D deficiency using vitamin D supplements could contribute to the prevention of cardiometabolic diseases.

The ultra-violet B (UVB) ray from the sun is the major and inexpensive source of vitamin D compare to other natural sources such as food, which contains a very small amount of vitamin D. Nevertheless; the prevalence of vitamin D deficiency is still high globally. In the United States, the prevalence of vitamin D deficiency was 25% to 57% as reported in the Third National Health and Nutrition Examination Survey (NHANES III) [27], whereas the prevalence of vitamin D deficiency was 28% in the Framingham Offspring Cohort study [19] and 47.8% in a study in Amsterdam [28]. This prevalence will remain elevated during the winter seasons, especially among the elderly and homebound geriatric patients [29-31]. Although vitamin D deficiency is common among people living in higher latitude countries, surprisingly high prevalence of vitamin D deficiency is also found in countries nearer to the equator which supposed to get sufficient sunlight all year round. Countries like Iran reported 61% of vitamin D deficiency [10], 64.6% in Bangkok, Thailand [32] and 66.3% in India [33]. These high rates of vitamin D deficiency and its association with cardiometabolic diseases suggesting an ever expanding importance of vitamin D roles in our health and well-being.

Malaysia is located in latitude 2° 30’ N and is blessed with sufficient sunshine all year round necessary for cutaneous synthesis of vitamin D. Khor et al. [34] however, reported that almost 35.3% of primary school children in Kuala Lumpur were vitamin D deficient, while approximately 70% of Malay adults in Kuala Lumpur were found to have vitamin D deficiency [35]. In addition, both studies found an inverse association between vitamin D status and obesity. These findings raise concerns as the prevalence of obesity in Malaysia is also on the rise [36]. Metabolic syndrome was found to be associated with vitamin D deficiency among Malay adults [35]. Therefore, there is a need for conclusive evidence from randomized controlled trials (RCT) to evaluate the roles of vitamin D in the primary prevention of cardiometabolic risks.

Therefore, we proposed to start a RCT with the following objectives:

i. To investigate whether vitamin D supplements can improve cardiometabolic risk factors such as blood pressure (BP), homeostasis model assessment insulin resistant (HOMA-IR), triglycerides (TG) and high-density lipoprotein cholesterol (HDL).

ii. To investigate whether vitamin D supplements can improve the vitamin D status of urban premenopausal women who are vitamin D deficient.

iii. To investigate whether vitamin D supplements can improve the health-related quality of life.

## Methods/design

### Study design

This is a double-blind, randomized placebo-control, parallel trial in which the participants will be randomized into 1:1 ratio.

### Setting

This study will be conducted at a public university in Kuala Lumpur, Malaysia. Kuala Lumpur is located near the equator at latitude 03° 09’N.

### Participants

All premenopausal women working in the public university will be invited for screening for their eligibility.

### Inclusion/exclusion criteria

All premenopausal women aged 30 years old and above with vitamin D deficiency; serum 25(OH)D level ≤ 20 ng/ml (or ≤ 50 nmol/l), will be invited to participate in this study. Premenopausal women are included as they will be at a lower risk for osteoporosis than post-menopausal women if they are vitamin D deficient.

Those with an abnormal level of serum parathyroid hormone (PTH) (>55 pg/ml) and serum calcium (> 10.4 mg/dl), known to have illnesses such as granuloma forming disorder (e.g. Tuberculosis), lymphoma, sarcoidosis or any type of cancers will be excluded. Women taking vitamin D supplements containing > 1000 IU/day or any forms of calcitriol (1,25(OH)_2_D_3_) will be excluded. Pregnant women or participants who were found to be pregnant during the study will also be excluded from the study.

### Sample size estimation

According to the study by Zittermann et al. [25], participants from the intervention arm with vitamin D supplementation was found to achieve a reduction in triglycerides of 0.19 ± 0.54 mmol/L and a change in mean for LDL-cholesterol of 0.19+1.03 mmol/L. Sample size calculation was conducted using OpenEpi version 2.3.1 Software. With a power of 80% and a two-sided 5% significant level, a minimum of 88 participants per arm is required using the results on triglycerides, while 146 participants is required with the results on LDL-cholesterol. In order to achieve adequate power, additions of 10% are added to 146 to account for possible attrition. Ultimately, the desired sample size for this study is 160 participants for each arm with a total of 320.

### Conduct of the study

The study will be divided into two phases: Phase I and Phase II.

#### Phase I

Premenopausal women who are interested to participate will be given brief introduction regarding the trial and offered for blood investigation for serum 25(OH)D, serum calcium, fasting blood insulin, fasting blood glucose, lipid profile and serum PTH as well as anthropometric and blood pressure measurements. Demographic data, sunlight exposure, physical activity and health-related quality of life questionnaires will also be administered at this stage. All the blood samples taken at this stage will be analysed and participants who meet the inclusion criteria will proceed to Phase 2.

#### Phase 2

**Figure 1:**
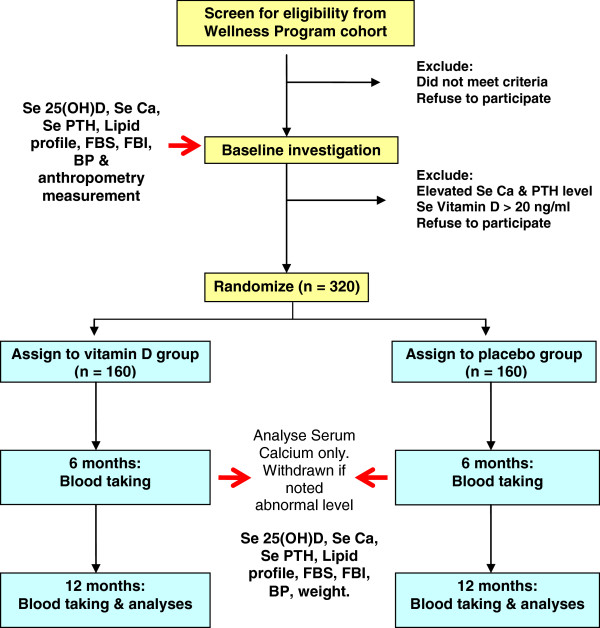
Recruitment flow chart.

Selected participants will be randomized into Vitamin D or placebo groups. Informed consent will be obtained from all participants at this stage. The intervention will be given for one year, and participants will be called for blood check at 6 months and 12 months after intervention completes (Figure 1). Table 1 shows the outcome measures and time for measurement.

**Table 1:** Outcome measures and time to be measured

**Measure**	**Months**		
	**0**	**6**	**12**
**Blood analysis**			
Serum 25(OH)D	**x**	**x**	**x**
Serum calcium	**x**	**x**	**x**
Serum PTH	**x**	**x**	**x**
Fasting blood glucose	**x**	**x**	**x**
Fasting blood insulin	**x**	**x**	**x**
Total cholesterol	**x**	**x**	**x**
Triglycerides	**x**	**x**	**x**
HDL-C	**x**	**x**	**x**
LDL-C	**x**	**x**	**x**
**Anthropometry and clinical assessments**			
Height, weight, BMI	**x**	**x**	**x**
Blood pressure	**x**	**x**	**x**
Waist circumference	**x**	**x**	**x**
**Questionnaires**			
Sociodemographics	**x**		
Medical and family history	**x**		
Dietary assessment	**x**		**x**
SF36 version 2	**x**		**x**
Sunlight exposure history	**x**		**x**
IPAQ	**x**		**x**

### Randomization

Eligible participants will be randomized into the active and placebo arm. The randomization sequence will be created using GraphPad Software with a 1:1 allocation by a staff (AC) with no involvement in the trial. The participant’s names will be matched with the random number sequence. The names of participants from intervention and placebo will be printed and pasted on to the tubes filled with either vitamin D powder or placebo. The tubes used will be identical for both active intervention and placebo, and allocation sequence data will be kept by AC in a secure place so that it cannot be accessed or influenced by anyone, including the researchers.

### Vitamin D intervention

The intervention consists of 50,000 IU of cholecalciferol (25(OH)D_3_) supplements with an average weight of 0.5 gram in the form of powder. The vitamin D supplements are purchased from DSM Nutritional products Ltd, Switzerland. Vitamin D supplement will be taken orally by the participants by diluting the powder into warm water before consumption. The consumption frequency will be once a week for 8 weeks (equivalent to 7142 IU/day) and then once a month for maintenance for 10 months (equivalent to 1667 IU/day). The consumption of 50,000 IU of vitamin D per week or per month, is considered to be safe as the tolerable upper intake level for vitamin D is 10,000 IU per day [37].

The placebo will be identical in taste, texture and colour with the active supplements and will be given the same manner of the active supplement. The vitamin D supplement and placebo will be packed by the Pharmacy Department of the University of Malaya Medical Centre (UMMC). Both the active supplements and placebo will be given to participants in a translucent pill container with only their names on the label. Labelling of the containers will be done by the same person (AC) doing the randomization and allocation sequence. The serum Vitamin D collected at different time points will only be analysed at the end of the trial. This will enable both the researchers and participants to be remained blinded throughout the trial.

To ensure compliance with the intervention, reminders about the intervention appointments will be sent regularly via phone messages, email or phone calls. For those who do not attend the intervention appointment, the researcher will visit them at their workplace to give them the active supplements or placebo personally.

### Blood sampling and methods of analysis

Venous blood samples will be taken by registered staff nurses using a sterile vacutainer needle and needle holder between 7.30 am and 9.30 am. All the participants will be asked to fast overnight for at least 8 hours before blood taking procedure. Blood serum will be used for the analysis of calcium (Ca), PTH, 25(OH)D_3_, fasting blood insulin and lipid profiles. The blood samples will be allowed clotting for about 30 minutes and centrifuged for 10 minutes at 2000 G at 4°C within 2 hours. All the blood serum will be stored at −80°C whilst waiting for analysis. Analysis of fasting blood glucose will be using blood plasma. Homeostasis model assessment of insulin resistance (HOMA-IR) will be used to evaluate insulin resistance. It can be calculated from a simple linear equation based on pairing FBS and Fasting Blood Insulin to establish a measure for insulin resistant: HOMA-IR = FBS (mmol/L) X Fasting Blood Insulin (IU per ml)/22.5. Radioimmunoassay analysis method (DiaSorin, Stillwater, MN) will be used to measure serum 25(OH)D. All tests and analysis will be carried out by the Clinical Diagnostic Laboratory, University Malaya Medical Centre.

### Anthropometry and blood pressure measurement

Blood pressure will be measured twice using a digital sphygmomanometer (OMRON HEM-907 model) in sitting position. The average of two measurements will be taken.

Measurement such as weight and height will be measured using calibrated digital weighing scales (Seca 808, Germany) and stadiometer (Seca, Germany) respectively. Body Mass Index (BMI) will be calculated from the formula of weight (kg)/height^2^(meters). Waist circumference will be measured using circumference measurement tape from the point midway between the iliac crest and costal margin (lower rib) in standing position and after expiration.

### Questionnaires administration

The questionnaires will contain two parts. Part 1 of the questionnaire enquires on socio demographic data, sunlight exposures, physical activity (International Physical Activity Questionnaires – IPAQ) and health-related quality of life questions (SF36® version 2 questionnaires). This questionnaire will be completed by participants at baseline.

The sunlight exposure questions will cover on history of sunlight exposure such as duration of outdoor activity in minutes per day as well as sunlight avoidance history such as usage of umbrella and sun block lotion. Participants are also enquired about their clothing style such as wearing long sleeves and hijab or veil. This questionnaire is obtained from a previous study done at the same setting [35].

The International Physical Activity Questionnaire (IPAQ) will be used to assess the physical activity patterns. It is a self-administered, 7-day period of IPAQ long form. We are using Malay version of IPAQ (IPAQ-M) and English version of IPAQ for the convenience of our participants in which they can choose to either answer the Malay or English version. IPAQ-M has been validated for use in Malaysian population [38].

SF-36® version 2 health survey will be used to assess participant’s health-related quality of life on physical and emotional wellbeing for participants with vitamin D deficiency and to assess if any improvements of health-related quality of life after intervention. Permission for usage of this questionnaire will be purchased from QualityMetric’s SF™. This health survey asks 36 questions measuring the functional health and well-being from participant’s point of view. It is a practical, reliable, and valid measure of physical and mental health. The SF-36 version 2 health survey will provide scores for each of the eight health domains, including the physical functioning, role-physical, bodily pain, general health, vitality, social functioning, role emotional and mental health together with psychometrically-based physical component summary (PCS) and mental component summary (MCS). The QualityMetric’s SF™ smart measurement system will be used to automatically calculate the scores. These questionnaires are available in Malay and English version, and it has been validated for use in Malaysian population by Sararaks et al. [39]. This questionnaire will be administered during screening stage and again at the end of the trial to see if there are any changes in quality of life after the intervention.

Second part of the questionnaire contains a 7-day dietary diary. Participants will be asked to give a detailed description of foods eaten and to estimate the amounts using natural or household measures (e.g. pieces, cups). An experienced dietitian will give instruction on how to fill in the diary. A list of instruction will also be provided in the diary booklet including examples. The dietitian will then convert the estimated amounts using a standardised protocol into weights as well as checking the completeness of the diary prior to data entry into The Nutritionist Pro software. Additional new foods and recipes will be added as required. Vitamin D composition in food will be obtained from The Nutritionist Pro™ Diet Analysis software (Axxya System LLC). After data entry, each record will be verified by a nutrition researcher and corrections will be made if any errors in data entry are found. All volunteers will be given a pre-addressed envelope for the return of the diary to the researcher within 2 weeks upon completion.

### Provision of results to participants

All participants at screening stage will be informed of their anthropometry, blood pressure measurement and blood results. Participants who fail to come to get their results will receive notification of all of their results together with advice about supplementation through mail where applicable.

On completion of the trial, participants will be informed of their current vitamin D status, and if they are taking the active or placebo dose. All participants in the placebo group will be given vitamin D supplements of 50,000 IU per week for 8 weeks and 50,000 IU per month for 4 months upon completion of the trial. Whereas participants taking active dose will be given 50,000 IU per month of vitamin D supplements for 3 months upon completion of the trial.

### Funding/ethics

This study was approved by the Medical Ethics Committee of University Malaya Medical Centre (reference number 907.22). This trial is funded by the University Malaya (Post Graduate Research Grant, PV080/2012A and University Malaya Research Grant, RG051/09HTM).

### Data handling and statistical analysis

We will use Microsoft Access to maintain the demographic data. To record the progress of each participant throughout the trial period, we will use a check box to allow for easy access and follow-up purposes. Raw data obtain from the questionnaire and blood results will be entered into SPSS software version 16.0 (SPSS Inc., 2009, Chicago, Illinois). The data will be cleaned and checked for coding errors. Preferably, data will be double-entered to check for duplication and outliers before commencement of statistical analysis.

For baseline characteristics, descriptive statistics will be used. Categorical data will be described using count, percentages and 95% confidence intervals when applicable. Exact test on proportions will be used to compare categorical variables since this is a comparative trial data. All numerical data will be checked for normality. For normally distributed data, mean with standard deviation, independent t-test will be used and analyse using SPSS 16.0. However, if the data is found to be non-normally distributed or have un-equal variances, exact (permutation) tests will be used. Exact tests analyses will be undertaken using *StatXact*_*®*_ 9 (Cytel Inc.).

The primary analysis will be performed to explore intervention’s effects on HOMA-IR, LDL-cholesterol, TG, HDL and BP These analyses will be following the “intention-to-treat” principle in the comparison between the intervention and the placebo group at 6 months and 12 months. Additionally secondary analyses will be a comparison of change of the vitamin D status and health-related quality of life. Level of statistical significance is preset at p < 0.05.

## Discussion

As suggested by M.F Holick [40], our study provides a dose of 50,000 IU weekly for 8 weeks and followed by 50,000 IU monthly for maintenance. This is to raise the blood level of 25(OH)D consistently above 30 ng/ml. It is coherent as suggested by the 2011 Endocrine Society Clinical Practice Guideline where they recommended that for adults aged 19 – 70 years, they require at least 1500 – 2000 IU/d of supplemental vitamin D. This method will be less costly, more convenient and will ensure compliance towards treatment than daily treatment.

The main purpose of this study is to evaluate the role of vitamin D supplements as the primary prevention measure for cardiometabolic risks. We hope to provide guidance for the general population, especially to people at risk such as elderly and women wearing hijab or veil to improve their vitamin D status by dietary intake or vitamin D supplements other than sunlight exposure. This study also will provide more evidence on the relationship between vitamin D and cardiometabolic risk. These results may be used to guide policy making decision in vitamin D supplementation or fortification for the public. It is well known that vitamin D intake improves individuals’ physical wellbeing. However, there is lacked of information on whether adequate vitamin D intake will improve mood and mental wellbeing as well. The relative question of whether vitamin D has a greater effect on quality of life need to be answered using clinical trials. We hope our results may answer these questions.

### Trial status

Upon submission, this study is in the process of participant’s recruitment.

## Abbreviations

25(OH)D: 25-Hydroxy-vitamin D; UVB: Ultra violet B; NHANES III: Third national health and nutrition examination survey; BP: Blood pressure; HOMA-IR: Homeostasis model assessment insulin resistant; TG: Triglycerides; HDL: High density lipoprotein cholesterol; LDL: Low density lipoprotein cholesterol; PTH: Parathyroid hormone; UMMC: University of Malaya Medical Centre; Ca: Calcium; IU: International unit; PCS: Physical component summary; MCS: Mental component summary; MI: Body mass index; IPAQ: International physical activity questionnaires; IPAQ-M: International physical activity questionnaires – Malay version; ANOVA: One-way analysis of variance

## Competing interests

The authors declare that they have no competing interests.

## Authors’ contributions

MR, FMM, RP, SS and ATTB participated in the design and coordination of the study. MR and FFM advised on statistical analysis. MR coordinated recruitment, participant management and data collection. MR drafted the manuscript. All authors reviewed the study protocol, made suggestions that improve the design and approved the final manuscript.

## Pre-publication history

The pre-publication history for this paper can be accessed here:

http://www.biomedcentral.com/1471-2458/13/416/prepub
